# Early-Life Exposure to Organic Chemical Pollutants as Assessed in Primary Teeth and Cardiometabolic Risk in Mexican American Children: A Pilot Study

**DOI:** 10.3390/ijerph22101494

**Published:** 2025-09-27

**Authors:** Vidya S. Farook, Feroz Akhtar, Rector Arya, Alice Yau, Srinivas Mummidi, Juan C. Lopez-Alvarenga, Alvaro Diaz-Badillo, Roy Resendez, Sharon P. Fowler, Hemant Kulkarni, Vijay Golla, Mahua Choudhury, Jane L. Lynch, Donna M. Lehman, Daniel E. Hale, Ralph A. DeFronzo, John Blangero, David E. Camann, Ravindranath Duggirala, Suman N. Challa

**Affiliations:** 1 Division of Human Genetics, University of Texas Rio Grande Valley, Brownsville, TX 78520, USAjohn.blangero@utrgv.edu (J.B.); 2 Department of Health and Behavioral Sciences, Texas A&M University-San Antonio, San Antonio, TX 78224, USA; rarya@tamusa.edu (R.A.); smummidi@tamusa.edu (S.M.); abadillo@tamusa.edu (A.D.-B.); rresendez@tamusa.edu (R.R.); hkulkarni@tamusa.edu (H.K.); dlehman@tamusa.edu (D.M.L.); rduggirala@tamusa.edu (R.D.); 3 Southwest Research Institute, San Antonio, TX 78238, USA; alice.yau@swri.org (A.Y.); david.camann@swri.org (D.E.C.); 4 Division of Population Health and Biostatistics, University of Texas Rio Grande Valley, Harlingen, TX 78550, USA; juan.lopezalvarenga@utrgv.edu; 5 Department of Medicine, University of Texas Health San Antonio, San Antonio, TX 78229, USA; fowlers@uthscsa.edu (S.P.F.); defronzo@uthscsa.edu (R.A.D.); 6 School of Public Health-San Antonio Campus, School of Public Health, Texas A&M University, College Station, TX 77843, USA; vijay.golla@tamu.edu; 7 Department of Pharmaceutical Sciences, College of Pharmacy, Texas A&M University, College Station, TX 77843, USA; drmc@tamu.edu; 8 Department of Pediatrics, University of Texas Health San Antonio, San Antonio, TX 78229, USA; lynchj2@uthscsa.edu; 9 Department of Pediatric Endocrinology and Diabetes, Penn State University, Hershey, PA 17033, USA; dhale2@pennstatehealth.psu.edu; 10 Department of Comprehensive Dentistry, University of Texas Health San Antonio, San Antonio, TX 78229, USA; challas@uthscsa.edu

**Keywords:** phthalate, acetaminophen, childhood obesity, diabetes, cardiometabolic risk

## Abstract

Early-life exposure to organic chemicals (OCs) may influence childhood obesity and associated cardiometabolic risk. These conditions have been shown to disproportionately affect minority populations such as Mexican Americans (MAs). However, information on the impact of organic chemicals on cardiometabolic risk in MA children is limited. Therefore, we conducted a pilot study to assess the extent to which exposure to organic chemicals influences cardiometabolic traits (CMTs) in MA children. We recalled 25 children from a previous study and collected 25 primary teeth from them. Chemical analyses of the teeth were performed using established protocols. Target analytes included acetaminophen (APAP); 3,5,6-trichloro-2-pyridinol (TCPy), 2-isopropyl-6-methyl-4-pyrimidinol (IMPy), diethyl phosphate (DEP), N,N-diethyl-m-toluamide (DEET), tris(2-butoxyethyl) phosphate (TBOEP), monoethyl phthalate (MEP), mono-n-butyl phthalate (MnBP), monoisobutyl phthalate (MiBP), monobenzyl phthalate (MBzP), mono-2-ethylhexyl phthalate (MEHP), mono-(2-ethyl-5-carboxypentyl) phthalate (MECPP), mono-(2-ethyl-5-oxohexyl) phthalate (MEOHP), and mono-(2-ethyl-5-hydroxyhexyl) phthalate (MEHHP). The organic chemicals most frequently detected in the teeth were APAP; the insect repellent DEET; plasticizers MnBP and MiBP; and the plasticizer-derived metabolite MEHP. These five analytes were included in association analyses with selected CMTs. After adjusting for covariate (age, sex, tooth-type) effects, we found significant (*p* < 0.05) positive correlations between MiBP and the following CMTs: fat mass, fasting insulin, and the homeostasis model of assessment-insulin resistance (HOMA-IR). Both MnBP and MEHP exhibited negative correlation with blood pressure measures and triglycerides, respectively. In addition, APAP showed a strong negative correlation with HDL-C (*p* = 0.009) and positive association with triglycerides (*p* < 0.10). These findings suggest a potential role for early-life exposures to organic chemicals in influencing cardiometabolic risk in MA children.

## 1. Introduction

Childhood obesity is associated with both short- and long-term inimical health consequences. Rapid weight gain during infancy tracks well into adulthood, increasing the risk of type 2 diabetes (T2D), cardiovascular disease (CVD), and metabolic syndrome (MS), which refers to the clustering of cardiometabolic abnormalities including obesity, insulin resistance, dyslipidemia and hypertension, as well as premature mortality and disability later in life [1,2,3,4,5,6]. The prevalence of childhood obesity has accelerated during the past few decades, and it has become a new pandemic, leading to a widespread global health crisis [7,8,9,10,11]. In the United States (US), disparities in obesity rates are significantly evident, with higher prevalence among children and adolescents from disadvantaged backgrounds. Obesity rates among Hispanic adults and children are dramatically higher than those among non-Hispanic whites [12]. For example, based on the National Health and Nutrition Examination Survey 2017-March 2020, among adults aged 20 years and above, obesity prevalence in non-Hispanic whites was 41.4% compared to 49.9% in non-Hispanic blacks, 45.6% in Hispanics, and 16.1% in non-Hispanic Asians; the prevalence among children and adolescents aged 2–19 years was highest among Hispanics (26.2%), followed by non-Hispanic blacks (24.8%), non-Hispanic white (16.6%), and non-Hispanic Asians (9.0%) [12]. Childhood obesity has multifactorial origins [13,14]. In addition to genetic determinants of obesity, dietary intake, sedentary lifestyle, stress, microbiota, social determinants, and other environmental factors contribute to the development of obesity [13,14,15,16,17,18,19,20,21,22,23,24,25,26,27,28]. These determinants act at multiple levels during the pre-, peri- and post-natal stages, and can induce lifelong and often irreversible derangements in the offspring’s adiposity and metabolism [29,30,31,32,33]. In addition, numerous studies have identified organic chemicals (OCs) as contributing factors for increased rates of obesity and metabolic syndrome [34,35,36,37]. By dysregulating energy metabolism and upregulating adiposity, these compounds act as obesogens (e.g., endocrine disruptors) [38,39,40]. Up to 20 different chemical classes are hypothesized to be obesogenic; these include persistent organic pollutants (POPs) and other compounds such as polychlorinated biphenyls (PCBs), hexachlorobenzene (HCB), dichlorodiphenyldichloroethylene (DDE), phthalates, bisphenol A (BPA), perfluoroalkyl substances (PFAS) and triclosan [39,41,42,43]. In addition, the OC acetaminophen (APAP) which is a widely used over-the-counter antipyretic and analgesic agent; and early-life exposures to APAP or its metabolites were found to be associated with childhood overweight or obesity [44,45,46].

Findings from several cross-sectional studies show positive association of POPs and OCs with T2D and obesity related traits in the general US population [37,47,48,49,50,51]. However, few longitudinal studies have examined the associations between early-life exposures to OCs and subsequent childhood obesity [46,52,53]. Current knowledge of exposures to low levels of OCs at critical periods of prenatal, perinatal, and postnatal development as they relate to childhood obesity and cardiometabolic risk is limited. Characterizing past exposures, especially during crucial periods, such as in utero and in early childhood, has been shown to be critical for assessing their impacts on health conditions later in life [54,55,56]. This being so, naturally shed primary teeth, due to their ability to sequester circulating semi-volatile organic compounds, offer a unique resource for measuring early-life exposures [54,55,56,57,58].

**Figure 1:**
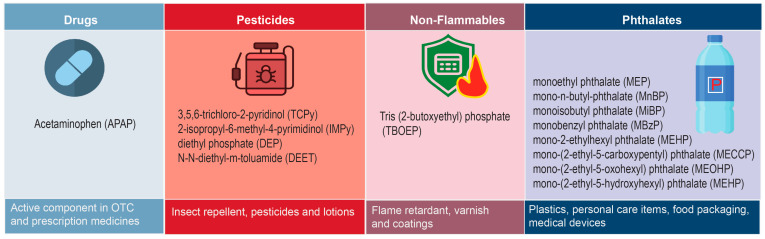
Target analytes: Metabolites of exogenous chemicals from four distinct environmental sources.

Early-life exposure to OCs could impact obesity and associated comorbid conditions such as T2D later in life, likely by altering energy metabolism and epigenetic regulation, with potential public health implications [59,60,61,62]. Therefore, we hypothesized that exposures to low levels of OCs during early life including the critical periods of prenatal, perinatal, and postnatal development, might be associated with obesity- and metabolic syndrome-related traits. To test this hypothesis, we carried out a pilot study, which measured the levels of 14 OCs in naturally shed primary teeth (Figure 1) from 25 Mexican American children and adolescents, to assess their association with childhood obesity and its related cardiometabolic traits (CMTs). These OCs represent common environmental and pharmaceutical exposures with relevance to potential disease risk, which were selected based on the published literature, particularly our own studies using established protocols that provided evidence that primary teeth can be valuable source of biomarkers of early-life exposures [63,64]. These children were participants in a previous study which revealed a high burden of overweight (53%), obesity (34%), prediabetes (13%), and metabolic syndrome (19%) [65].

## 2. Materials and Methods

### 2.1. Study Population

The data used for this study were related to 25 participants of the San Antonio Family Assessment of Metabolic Risk Indicators in Youth (SAFARI) study. SAFARI is a community-based family study designed to identify signs of metabolic syndrome and future disease risk in MA children and adolescents in San Antonio, TX and surrounding areas, and to examine their genetic basis [65]. As part of the SAFARI study, 673 children and adolescents, aged 6–17 years old, were recruited from large, predominantly lower-income MA families at increased risk of obesity and T2D, whose adult family members had previously participated in one of three community-based genetic epidemiological studies in San Antonio, TX: the San Antonio Family Diabetes/Gallbladder Study (SAFDGS), the San Antonio Family Heart Study (SAFHS), and the Veterans Administration Genetic Epidemiology Study (VAGES) [66,67,68]. Several demographic, anthropometric, and cardiometabolic traits were measured and environmental covariate data were collected as part of the SAFARI study. For this pilot study, the parents of SAFARI study participants were contacted by sending the project information (i.e., recruitment materials sent via mail) to collect primary teeth. There were no attempts to select children based on any clinical criteria (e.g., disease condition), and the children of the first 25 responders (i.e., parents of 25 children with available primary teeth) to the project request were recruited to assess the association of early-life exposure to low levels of OCs with risk of childhood obesity and related CMTs. All research procedures were approved by the Institutional Review Board of the University of Texas Health San Antonio, San Antonio, TX. Prior to the initiation of the study, informed written consent was obtained from either or both parents or the guardian of each child, and signed assent was obtained from children ≥ 7 years old.

### 2.2. Phenotypic Data

Data on obesity/CMTs used for this study were obtained using standard protocols as previously described by Fowler et al. 2013 [65]. Briefly, the following 13 CMTs were considered for this study: body mass index (BMI: weight [kg] divided by height [m] squared), waist circumference (WC), fat mass (FM: assessed by dual-energy-X-ray absorptiometry [DXA Hologic]), systolic (SBP) and diastolic blood pressure (DBP), fasting insulin (FI), glucose (FG), homeostasis model of assessment-insulin resistance (HOMA-IR, measured using FI and FG levels), high-density lipoprotein cholesterol (HDL-C), triglycerides (TG), aspartate aminotransferase (AST), alanine aminotransferase (ALT), and aspartate aminotransferase/alanine aminotransferase ratio (AST/ALT ratio).

### 2.3. Tooth Preparation

Twenty five tooth crowns were pulverized and analyzed for the following 14 target analytes representing four distinct environmental sources (Figure 1): acetaminophen (APAP), 3,5,6-trichloro-2-pyridinol (TCPy), 2-isopropyl-6-methyl-4-pyrimidinol (IMPy), diethyl phosphate (DEP), N,N-diethyl-m-toluamide (DEET), tris(2-butoxyethyl) phosphate (TBOEP), monoethyl phthalate (MEP), mono-n-butyl phthalate (MnBP), monoisobutyl phthalate (MiBP), monobenzyl phthalate (MBzP), mono-2-ethylhexyl phthalate (MEHP), mono-(2-ethyl-5-carboxypentyl) phthalate (MECPP), mono-(2-ethyl-5-oxohexyl) phthalate (MEOHP), and mono-(2-ethyl-5-hydroxyhexyl) phthalate (MEHHP), using modified methods from Camann, Schultz et al. [63]. Each primary tooth crown type was identified by the study dentist who is part of this study (SC). Briefly, the crown of each exfoliated tooth was severed at the cementoenamal junction; pulp was scraped out, and any fillings, attached roots and/or cavities crown were removed with dental post-extraction tools (scalpel, engraving tool and heatless wheel). Each crown sample was placed in 1 mL of dichloromethane (DCM) and gently swirled to remove possible contamination during sample collection and preparation. The DCM-washed tooth crown was then pulverized with a mortar and pestle to a fine powder, and the powder weighed. The DCM used for the wash was retained as a quality measure to evaluate external tooth contamination.

### 2.4. Extraction and Analysis by Liquid Chromatograpgy-Tandem Mass Spectrometry (LC/MS/MS)

To determine target analytes approximately 25 mg of pulverized tooth sample was used in the extraction procedure [63]. Each pulverized tooth aliquot was spiked with 50 μL of labeled internal standards (labeled as APAP and MEHP surrogate) before extraction. To enhance the extraction efficacy of analytes, each aliquot of tooth was extracted under neutral and acidic conditions. The first neutral extraction employed sonication with 1 mL acetonitrile for 1 h followed by centrifugation at 3000 rpm for 5 min. The supernatant was concentrated to 25 μL and transferred to a new vial. For acidic extraction 50 μL of glacial acetic acid was added to each tooth sample aliquot and equilibrated overnight. A second extraction by repeating the acetonitrile sonication extraction was performed. The concentration of the target analytes in each powdered tooth extract was determined by high performance liquid chromatography (HPLC)/MS/MS in multiple reaction monitoring (MRM) mode with electrospray ionization. It should be noted that no biomarker validation study of OC measurement in primary teeth as we suggested [63] has yet been performed.

### 2.5. Statistical Analysis

The descriptive statistics and assessment of the bivariate Pearson correlations between the OCs and obesity/CMTs were carried out using the Statistical Package for the Social Sciences (SPSS) software(IBM SPSS Statistics 25). Prior to performing the correlation analyses, all obesity/CMTs were adjusted for the covariate effects of age and sex, for consistency and comparability across the models. The trait-specific residuals (i.e., residual phenotype), after accounting for the influences of age and sex through a regression analysis, were inverse normalized to address the issue of non-normality. Regarding the OCs, a similar approach was applied, but tooth type (i.e., incisor, canine, first molar, and second molar as dummy variables) was included as an additional covariate to account for differential exposure histories captured by different teeth. The trait-specific residuals (i.e., residual phenotype), after controlling for the influences of age, sex, and tooth type through a regression analysis, were inverse normalized to address the issue of non-normality. The trait-specific inverse normalized residual values were then used to obtain the bivariate Pearson correlations between OCs and obesity/CMTs (i.e., residual phenotypes). Given the pilot nature of this study, all significant (*p* ≤ 0.05) and suggestive (*p* < 0.10) correlations were considered for discussion.

## 3. Results

The demographic and cardiometabolic characteristics of these children (Girls: 56%, mean age: 10.5 years, and mean BMI: 22.4) are shown in Table 1. The distributions and concentrations of the selected 14 targeted OCs obtained from the 25 teeth are summarized in Table 2. The monobutyl ester phthalate metabolites MnBP and MiBP were detected in every tooth, often at high levels. Although quantification was obscured by moderate levels in some blanks, resulting in not reported (NR) values, the insect repellant DEET and the diethylhexyl phthalate metabolite MEHP were detected in nearly every tooth, often at high levels. The diethyl phthalate metabolite MEP was detected at high levels in a few teeth, though quantification was obscured by high levels in all blanks, resulting in many NR values. The secondary oxidative DEHP phthalate metabolites (MECCP, MEOHP, and MEHHP), which are present at higher levels than MEHP in urine, were rarely detected in these teeth, although a clear explanation for this observation is unknown. As reported in Table 2, the specific metabolite TCPy of the organophosphate insecticide chlorpyrifos, the analgesic APAP, the organophosphate flame retardant TBOEP, the specific metabolite IMPy of the organophosphate insecticide diazinon, and monobenzyl phthalate (MBzP) were occasionally detected in these teeth.

For data analysis, the five most frequently detected OCs in the LC/MS/MS analysis of the 25 teeth were considered: the insect repellant DEET; MnBP, MiBP, and MEHP, which are metabolites of phthalates commonly used as plasticizers; and the analgesic APAP. We replaced the non-detected values of APAP with 0.25 ng/g, which is slightly below the lowest detected APAP concentration (0.33 ng/g). The Pearson correlations, significant or suggestive, between the five most frequently detected OCs and obesity/CMTs are reported in Table 3. Of the five OCs considered for the analysis, DEET is not associated with obesity/CMTs. As shown in Table 3, of the three phthalate metabolites considered for the analyses, MiBP positively and significantly correlated with three CMTs: FM (r = 0.496, *p* = 0.012), FI (r = 0.594, *p* = 0.002), and HOMA-IR (r = 0.560, *p* = 0.004). MnBP exhibited significant negative correlations with both blood pressure measures: SBP (r = −0.428, *p* = 0.033) and DBP (r = −0.537, *p* = 0.006), while MEHP was found to be negatively and significantly associated with TG (r = −0.485, *p* = 0.019). Interestingly, APAP showed a negative and significant association with HDL-C (r = −0.518, *p* = 0.009), and a positive association with TG (r = 0.399) that was suggestive (*p* = 0.059) in nature. Its positive association with ALT (r = 0.471) was significant (*p* = 0.020), while its positive association with AST was suggestive (r = 0.376, *p* = 0.070).

## 4. Discussion

Environmental influences in early life including the pre-, peri-, and post-natal periods can be detrimental in shaping an individual’s developmental trajectories [69]. This is well understood in the context of obesity and diabetes, where exposures to POPs and OCs during the early stage of life are linked to the burden of adult obesity and related diseases [38]. Measuring low levels of toxic chemicals and exposures during the critical span of prenatal, perinatal, and postnatal growth phases has been an emerging field of research investigations. By exploiting the primary teeth as a biological tool for examining the accumulation of traces of chemicals, we assessed the exposures captured by them during the early developmental stages and analyzed their influence on obesity and CMTs in Mexican American children. The use of exfoliated teeth as a biomarker for past chemical exposure is promising on many counts. First, they are expected to capture early-life chemical exposures in a continuum starting from the second trimester in utero through the stage where primary teeth are shed. Second, they stably store chemicals with decade-long half-lives, unlike such common biological matrices such as urine and blood, which have short half-lives for some chemicals; and, they are non-invasive and easy to collect [57,58,63,70]. Despite their promising nature as a source of biomarkers, primary teeth are relatively understudied biospecimens for organic chemical exposures studies. Our results demonstrated that specific organic compounds can be detected in primary teeth of children. The target analytes included APAP, an active component in many over-the-counter (OTC) and prescription medicines, and metabolites of exogenous chemicals that are ubiquitous in the environment. Monobutyl ester phthalate metabolites MnBP and MiBP, the diethylhexyl phthalate metabolite MEHP, and DEET were detected in every tooth, often at high levels. These are consistent with the previous report from Camann et al. [63]. In this pilot study we found that early-life exposure to phthalate metabolites was associated with several markers of cardiometabolic risk in children. Additionally, we observed an association between APAP and lipids and liver function markers. To our knowledge this study is the first to examine the relationship between early-life exposures measured from primary teeth and several cardiometabolic traits in MA children. In our study, APAP concentration was significantly negatively associated with HDL-C (*p* = 0.009) and exhibited a suggestive positive correlation with triglycerides (*p* = 0.059). In spite of its short half-life (2 to 2.5 h), APAP readily accumulates in pediatric patients after repeated therapeutic doses [71,72]. At correct dosage, APAP use is considered safe. However, APAP over-dosage is a leading cause of drug-induced liver injury worldwide [73,74,75]. The current study revealed that APAP was significantly and positively correlated with alanine aminotransferase (ALT) levels (*p* = 0.020), and that aspartate aminotransferase (AST) exhibited a suggestive positive association with APAP (*p* = 0.07). Results from murine models and human volunteers have established the hepatotoxicity consequences of APAP overdose due a wide variety of complex and interrelated pathophysiological processes such as mitochondrial oxidative and ER stress, autophagy and inflammation [73,76]. Elevated levels of long-chain acylcarnitines, triglycerides and free fatty acids in the serum of mice and humans due to APAP-induced mitochondrial dysfunction have also been reported [77,78]. HDL-C levels were significantly higher in patients with acute liver failure caused by APAP intoxication [79]. Maternal use of APAP in pregnancy has been associated with autism spectrum disorder [80], poor communication, behavioral problems [81] and asthma in their offspring [82]. Investigations of early-life APAP exposure and obesity-related outcomes in humans are scarce. To date, a few studies have found a relationship between early-life exposure to APAP and obesity. One such study identified activation of cannabinoid receptors by APAP may promote weight gain as reviewed in [83]. Several animal studies have highlighted the important role of cannabinoid receptor activation in the hypothalamus and limbic system in promoting hunger, food intake, and increased body weight [83]. Murphy et al. showed that APAP use in infancy or childhood may be causally related to increased BMI [44]. Sorrow et al. reported a positive association between APAP metabolites at birth and childhood obesity [46].

We found higher MnBP, MiBP, and MEHP concentrations in the SAFARI children’s teeth compared to those detected in a previous study [64], which may be due to the relatively low socioeconomic status of the SAFARI study participants [84]. Of these three most-detected phthalate metabolites in our study, MiBP showed a significant positive correlation with key indicators of obesity and metabolic syndrome, including fat mass (*p* = 0.012), fasting insulin (*p* = 0.002), and insulin resistance (HOMA-IR; *p* = 0.0004). MnBP exhibited strong association with lower diastolic blood pressure (*p* = 0.006) and to a lesser extent, with lower systolic blood pressure (*p* = 0.033). Inverse association with triglyceride (0.019) was noted for MEHP.

While exposure studies on phthalate metabolites in adults have established direct associations with adverse health outcomes such as obesity, cardiometabolic traits, and glucose metabolism disorders [52,53,85,86,87,88,89,90,91], childhood and prenatal studies with phthalate exposure are now in focus. Low-molecular-weight metabolites such as MiBP, MnBP and MEP have been associated with childhood obesity [60,92], whereas and high-molecular-weight phthalate metabolites, with adult obesity [60,92,93]. Although, exposure to MiBP has been reported to increase the odds for elevated BMI, waist circumference and percent body fat [94], conflicting findings were reported by [95,96], who noted no significant association between phthalate metabolite and markers of childhood obesity. The multiracial/ethnically diverse study population may be the reason for the reported inconsistency. Consistent with our results, metabolites of DEHP have been associated with an increase in fasting glucose, insulin, and insulin resistance, and reduction in glucose control in adolescents, adults, and elderly adults [86,89,97,98]. Studies assessing early-life phthalate exposures in relation to insulin resistance have also yielded contradictory outcomes; early-life exposure to phthalates associated with increased HOMA-IR was found in one study [99] but not in the other [100]. Consistent with our findings, studies on the impact of phthalate exposure on lipid metabolism have shown an association of MEHP with increased TG levels [99,101]. In addition, the association of early-life exposure to phthalates and changes in blood pressure shows varying outcomes. Several studies have linked MiBP exposure to elevated systolic blood pressure in children [89,102,103]. However, as found in our study, recent evidence indicates modest reductions in systolic and diastolic BP [52,53,104].

Multiple mechanisms may be involved in linking phthalates to increased body weight, adiposity and cardiovascular risk. Phthalates are known to interfere with peroxisome proliferator-activated receptors (PPARs), key regulators of adipogenesis and energy storage [105,106]. Phthalates can modulate adipocyte differentiation and release of leptin by activating PPAR-y [107]. Laboratory studies have found that phthalate metabolites increase cytokine production [108], while biomarkers of phthalate exposure have been associated with increases in serum markers of inflammation and oxidative stress in adults. Recent findings suggest that phthalates may produce increases in low-grade albuminuria in children, a marker of vascular dysfunction associated with chronic kidney and CVD risk [109]. Moreover, epigenetic alterations including DNA-methylation modifications are suggested to be the modifiers of perinatal programming leading to later-life diseases including obesity. Also, several OCs, including phthalates, have been associated with modifications in DNA-methylation and microRNA expression [110]. These studies suggest multiple mechanisms by which phthalates and other OCs may exert their roles as modifiers of prenatal programming and thus lead to future development of obesity.

Given the exploratory nature of this pilot study, there are several inherent limitations that need to be considered. A major limitation is the small sample size used, representing a subset of an existing study. However, the collection of primary teeth at a large scale in a given study population can be challenging. Hence, the findings reported here are limited in generalizability and should be interpreted with caution. In addition, we are not able to establish causal relationships between the examined variables due to the limitations of the cross-sectional study design. Furthermore, as we previously stated [62], the developmental timing of exposure to the OCs measured in this study within the window between mineralization and tooth loss has not been established. This potentially could weaken their correspondence with cardiometabolic risk. Despite these limitations, our findings do offer valuable insights that can be leveraged for future studies. In fact, we plan to conduct validation studies in the near future to increase the reliability and generalizability of our findings. Also, similar studies in ethnically diverse populations would help to assess the applicability of our study findings to a wider population. Nevertheless, our study has some notable strengths. The approach of using primary teeth as a source of biomarkers to assess early-life exposure to organic chemicals is a major strength. The pervasive presence of OCs, POPs, and EDCs, in the primary teeth reflecting study-specific early-life environmental exposures provides a unique opportunity for future in-depth research. Furthermore, this study precisely identified certain chemical exposures significantly associated with cardiometabolic risk in children and adolescents, underscoring the potential long-term health implications of early-life environmental exposures.

## 5. Conclusions

This is the first study to use primary teeth as a biomarker for pre-, peri-, and post-natal low-level exposure to organic chemicals, to explore their potential role in the occurrence of obesity and associated cardiometabolic risk in Mexican American children and adolescents. Despite the small sample size, our findings suggest that early-life exposure to phthalates may exert adverse effects on children’s heath, increasing the risk of childhood obesity and its comorbid conditions. Additional studies with a larger study population are necessary to confirm these findings, as well as to determine the molecular bases of the associations observed in this study, and to develop effective intervention strategies to reduce exposure to phthalates. Thus, the findings from this study suggest the need for public awareness regarding the ill effects of organic chemicals such as phthalates that are present in commonly used products (e.g., toys, cosmetics, kitchen items, etc.). Such public health efforts are essential to reduce the harmful effects of early-life exposures to organic chemicals that have direct relevance to childhood obesity and its associated cardiometabolic risk [60,111].

## Figures and Tables

**Table 1:** Characteristics of 25 Study Participants.

Variable *	N	Mean ± SD or %
Girls	25	56
Age (years)	25	10.48 ± 3.61
Body mass index [BMI] (kg/m^2^)	25	22.36 ± 6.62
Waist circumference [WC] (mm)	24	750.69 ± 190.44
Systolic blood Pressure [SBP] (mmHg)	25	103.04 ± 6.07
Diastolic blood pressure [BP] (mmHg)	25	62.56 ± 4.85
HDL cholesterol [HDL-C] (mg/dL)	24	45.63 ± 5.91
Triglycerides [TG] (mg/dL)	23	69.91 ± 20.07
Fasting glucose [FG] (mg/dL)	25	88.69 ± 7.29
Fasting insulin [FI] (µIU/mL)	24	13.57 ± 6.98
Fat mass [FM] (kg)	25	11.02 ± 15.36
HOMA-IR	24	1.96 ± 0.95
Aspartate aminotransferase [AST] (unit/L)	24	24.00 ± 6.40
Alanine aminotransferase [ALT] (unit/L)	24	16.99 ± 8.18
AST/ALT ratio	24	2.17 ± 2.21

* HOMA-IR = Homeostasis model of assessment-insulin resistance and AST/ALT ratio = Aspartate aminotransferase/Alanine aminotransferase ratio.

**Table 2:** Concentration Distributions of Organic Chemicals in a Primary Tooth of 25 Study Participants.

Analyte	DL ^@^	Detections		Concentration (ng/g)		
	ng/g	No. ^@^	%	Median	75th %ile ^@^	Max. ^@^
Acetaminophen (APAP)	1	9	36		1.7	9.6
3,5,6-trichloro-2-pyridinol (TCPy)	1	6	24		0.8	62.2
2-isopropyl-6-methyl-4-pyrimidinol (IMPy) ^#^	1	2 ^#^	8			5
Diethyl phosphate (DEP)	5	0	0			
N,N-diethyl-m-toluamide (DEET) *^#^	17	24 ^#^	100	45	289	946
Tris(2-butoxyethyl) phosphate (TBOEP) *^#^	3	2 ^#^	12			71.5
Monoethyl phthalate (MEP) *^#^	1200	4 ^#^	100 ^&^			4000
Mono-*n*-butyl phthalate (MnBP) *	26	25	100	715	1670	19,700
Monoisobutyl phthalate (MiBP) *	13	25	100	186	622	5680
Monobenzyl phthalate (MBzP)	5	1	4			26
Mono-2-ethylhexyl phthalate (MEHP) *^#^	12	21 ^#^	100	158	799	2610
Mono-(2-ethyl-5-carboxypentyl) phthalate (MECPP)	5	0	0			
Mono-(2-ethyl-5-oxohexyl) phthalate (MEOHP)	5	0	0			
Mono-(2-ethyl-5-hydroxyhexyl) phthalate (MEHHP)	5	1	4			30.5

^#^ The concentrations presented in this table were adjusted after subtracting the blank values. It excludes not reported concentration values for MEP (21 teeth), MEHP (4 teeth), TBOEP (8 teeth), DEET (1 tooth), and IMPy (1 tooth); ^@^ DL = Detection Limit, No. = Number of teeth in which the analyte was detected above the DL, % = Percentage of detections above the DL, where teeth with not reported values are excluded, 75th %ile = 75th Percentile, and Max. = Maximum; ^&^ MEP was excluded from the analysis due many NR values (please see text); * Concentration after subtraction of larger of matrix and solvent blanks.

**Table 3:** Correlations * between Organic Chemicals and Obesity and Cardiometabolic Traits in 25 Study Participants.

Variable ^#^	FM	HDL-C	TG	SBP	DBP	FI	HOMA-IR	AST	ALT
APAP	*0.278*	**−0.518**	*0.399*	*0.342*	*0.197*	*0.279*	*0.301*	*0.376*	**0.471**
	*(0.179)*	**(0.009)**	*(0.059)*	*(0.940)*	*(0.345)*	*(0.187)*	*(0.154)*	*(0.07)*	**(0.02)**
DEET ^&^	*0.149*	*−0.059*	*0.010*	*0.114*	*−0.127*	*0.181*	*0.215*	*−0.152*	*0.035*
	*(0.476)*	*(0.786)*	*(0.964)*	*(0.586)*	*(0.546)*	*(0.396)*	*(0.312)*	*(0.479)*	*(0.872)*
MnBP	*−0.212*	*0.025*	*0.072*	**−0.428**	**−0.537**	*−0.161*	*−0.183*	*−0.002*	*−0.199*
	*(0.308)*	*(0.908)*	*(0.745)*	**(0.033)**	**(0.006)**	*(0.453)*	*(0.393)*	*(0.993)*	*(0.350)*
MiBP	**0.496**	*−0.092*	*−0.285*	*0.030*	*−0.037*	**0.594**	**0.56**	*−0.012*	*0.227*
	**(0.012)**	*(0.669)*	*(0.188)*	*(0.887)*	*(0.862)*	**0.002**	**0.004**	*(0.957)*	*(0.287)*
MEHP	*0.174*	*0.115*	**−0.485**	*−0.120*	*−0.212*	*0.209*	*0.195*	*−0.194*	*0.075*
	*(0.405)*	*(0.593)*	**(0.019)**	*(0.569)*	*(0.310)*	*(0.326)*	*(0.361)*	*0.363*	*(0.726)*

* Pearson correlation coefficients and the values within the parentheses represent *p*-values. Significant correlations (*p* ≤ 0.05; shown in bold), suggestive correlations (*p* < 0.10) and non-significant correlations (shown in italics) are shown; ^#^ APAP, acetaminophen; MnBP, Mono-n-butyl phthalate; MiBP, Monoisobutyl phthalate; MEHP, Mono-2-ethylhexyl phthalate; FM, Fat mass; HDL-C, High-density lipoprotein cholesterol; TG, Triglycerides; SBP, Systolic blood pressure; DBP, Diastolic blood pressure, FI, Fasting insulin; HOMA-IR, Homeostasis model of assessment-insulin resistance; AST, Aspartate aminotransferase; ALT, Alanine aminotransferase; ^&^ The results of DEET (N,N-diethyl-m-toluamide) are shown for the selected variables in the Table although it was not associated with any obesity/CMTs examined in this study (please see text).

## Data Availability

The data presented in this study are available on request from the corresponding author due to privacy and ethical issues.

## Abbreviations

The following abbreviations are used in this manuscript:

ALT	Alanine aminotransferase
APAP	Acetaminophen
AST	Aspartate aminotransferase
AST/ALT ratio	Aspartate aminotransferase/Alanine aminotransferase ratio
BMI	Body mass index
BPA	Bisphenol A
CMTs	Cardiometabolic traits
CVD	Cardiovascular diseases
DBP	Diastolic blood pressure
DCM	Dichloromethane
DDE	Dichlorodiphenyldichloroethylene
DEET	N,N-diethyl-m-toluamide
DEP	Diethyl phosphate
DL	Detection limit
DXA	Dual X-ray Absorptiometry
EDC	Endocrine disrupting chemicals
FG	Fasting glucose
FI	Fasting insulin
FM	Fat mass
HDL-C	High-density lipoprotein cholesterol
HCB	Hexachlorobenzene
HOMA-IR	Homeostasis model of assessment-insulin resistance
HPLC/MS/MS	High-performance liquid chromatography/tandem mass spectrometry
IMPy	2-isopropyl-6-methyl-4-pyrimidinol
MAs	Mexican Americans
MBzP	Monobenzyl phthalate
MECPP	Mono-(2-ethyl-5-carboxypentyl) phthalate
MEHHP	Mono-(2-ethyl-5-hydroxyhexyl) phthalate
MEHP	Mono-2-ethylhexyl phthalate
MEOHP	Mono-(2-ethyl-5-oxohexyl) phthalate
MEP	Monoethyl phthalate
MiBP	Monoisobutyl phthalate
MnBP	Mono-n-butyl phthalate
MS	Metabolic syndrome
NR	Not reported
OCs	Organic chemicals
OTC	Over the counter
PCBs	Polychlorinated biphenyls
PFAS	Perfluoroalkyl and polyfluoroalkyl substances
POPs	Persistent organic pollutants
SAFARI	San Antonio Family Assessment of Metabolic Risk Indicators in Youth
SAFDGS	San Antonio Family Diabetes/Gallbladder Study
SAFHS	San Antonio Family Heart Study
SBP	Systolic blood pressure
SPSS	Statistical Package for the Social Sciences
T2D	Type 2 diabetes
TBOEP	Tris(2-butoxyethyl) phosphate
TCPy	3,5,6-trichloro-2-pyridinol
TG	Triglycerides
VAGES	Veterans Administration Genetic Epidemiology Study
WC	Waist circumference
